# Bilateral acute renal cortical necrosis after a dog bite: case report

**DOI:** 10.1186/s12879-021-05901-6

**Published:** 2021-02-27

**Authors:** Simon A. Amacher, Kirstine K. Søgaard, Coralie Nkoulou, Raoul Sutter, Maja Weisser, Sandra S. Zingg, Adrian Egli, Alexa Hollinger, Martin Siegemund

**Affiliations:** 1 Intensive Care Medicine, University Hospital Basel, Basel, Switzerland; 2 Division of Bacteriology and Mycology, University Hospital Basel, Basel, Switzerland; 3 Applied Microbiology Research, Department of Biomedicine, University of Basel, Basel, Switzerland; 4 Department of Clinical Research, University of Basel, Basel, Switzerland; 5 Division of Infectious Diseases and Hospital Epidemiology, University Hospital Basel, Basel, Switzerland

**Keywords:** Case report, Reverse rim sign, *Capnocytophaga canimorsus*, Dog bite, Acute kidney injury

## Abstract

**Background:**

*Capnocytophaga canimorsus* is a Gram-negative capnophilic rod and part of dogs/cats’ normal oral flora. It can be transmitted by bites, scratches, or even by contact of saliva with injured skin. Asplenic patients and patients with alcohol abuse are at particular risk for fulminant *C. canimorsus* sepsis. However, also immunocompetent patients can have a severe or even fatal infection. This is the first case of a severe *C. canimorsus* infection in an immunocompromised host complicated by acute renal cortical necrosis with a “reverse rim sign” in contrast-enhanced computed tomography on hospital admission.

**Case presentation:**

We report the case of a 44-year functionally asplenic patient after an allogeneic stem cell transplantation, who presented with septic shock after a minor dog bite injury 4 days prior. Because of abdominal complaints, epigastric pain with local peritonism, and radiological gallbladder wall thickening, an abdominal focus was suspected after the initial work-up. The patient underwent emergent open cholecystectomy, but the clinical suspicion of abdominal infection was not confirmed. Septic shock was further complicated by cardiomyopathy and disseminated intravascular coagulation. As a causative pathogen, *C. canimorsus* could be isolated. The clinical course was complicated by permanent hemodialysis and extensive acral necrosis requiring amputation of several fingers and both thighs.

**Conclusion:**

We present a severe case of a *C. canimorsus* infection in a functionally asplenic patient after a minor dog bite. The clinical course was complicated by septic shock, disseminated intravascular coagulation, and the need for multiple amputations. In addition, the *rare* form of acute *renal* failure - bilateral acute renal cortical necrosis – was visible as “reverse rim sign” on computed tomography scan. This case is an example of the potential disastrous consequences when omitting pre-emptive antibiotic therapy in wounds inflicted by cats and dogs, particularly in asplenic patients.

## Background

Acute kidney injury occurs in up to half of all septic patients [1, 2] and carries a significant risk of end-stage kidney disease with the frequent requirement of chronic renal replacement therapy [3].

Acute renal cortical necrosis (ARCN) is an uncommon form of acute kidney injury in states of shock and is the result of direct toxic damage to the glomerular endothelium, as well as microthrombi resulting from sepsis [4, 5]. In contrast-enhanced computed tomography (CT), the non-perfusion of the renal cortices due to necrosis results in a non-enhancement in contrast to adequately enhancing renal medullae mimicking a retrograde renography [4]. This CT finding is pathognomonic for ARCN and usually results in permanent kidney injury [6].

*Capnocytophaga canimorsus* is a slow-growing, capnophilic, facultative anaerobic Gram-negative rod. It was first described in 1977 as a dysgonic fermenter 2 (DF-2), and in 1989 taxonomically classified as *C. canimorsus* [7, 8]. Although *C. canimorus* is part of the normal oral flora in up to two-thirds of cats and dogs [9], only three serovars (A-C) cause invasive disease in humans [10]. Its primary mode of transmission to humans is by animal bites, but it can even be transmitted by licks of damaged skin and scratches [11, 12]. Immunocompromised patients are particularly vulnerable to fulminant *C. canimorsus* sepsis, but also immunocompetent patients may experience severe clinical courses with high morbidity and mortality [12, 13]. In the sub-group of immunocompromised patients, asplenic patients and patients with alcoholism are particularly vulnerable to *C. canimorus*. In two scoping reviews of published *C. canimorsus* cases, the most frequent clinical entities were sepsis complicated by septic shock, meningitis, multiple organ failure, peripheral gangrene, and DIC with a high case fatality rate of up to 56% [12, 13] Table 1 gives an overview of the variety of possible clinical presentations of *C.canimorsus.*

**Table 1:** Selection of clinical presentations of *C.canimorsus* infections

	Main clinical feature	Age/gender	Transmitting animal	Evidence of immunodeficiency	Survival to hospital discharge
Barry et al. [14]	ruptured mycotic common iliac aneurysm	86 years/female	dog	no	yes
Ashley et al. [15]	aortitis	80 years/female	dog	no	yes
Keshava et al. [16]	septic shock	42 years/female	dog	no	no
Terashima et al. [17]	hemophagocytic lymphohistiocytosis	58 years/male	dog	no	yes
Bering et al. [18]	purulent meningitis	67 years/male	cat	chronic lymphocytic leukemia	yes
Klein et al. [19]	septic gonarthritis	66 years/male	dog	no	yes
Squire et al. [20]	infective endocarditis	76 years/female	dog	no	yes
Tani et al. [21]	disseminated intravascular coagulation	62 years/female	dog	no	yes
Papadaki et al. [22]	endophthalmitis	60 years/male	dog	no	yes
Nelson et al. [23]	vertebral osteomyelitis/discitis	31 years/male	dog	no	yes
Ehrbar et al. [24]	myocardial infarction	52 years/male	dog	alcoholism	yes

## Case report

We recently treated a 44-year-old Caucasian woman who presented to a regional hospital with abdominal complaints and pyrexia for the last 24 h. Past medical history revealed an allogeneic stem cell transplantation 12 years ago due to acute lymphoblastic leukemia, which was in full remission. At the time of infection, the patient had no ongoing immunosuppressive treatment. However, there was evidence of transplantation related functional asplenia by the repetitive detection of Howell-Jolly bodies in peripheral blood smears and a CT-radiographic small, atrophic spleen with a length of 3.8 cm. Four days prior to presentation, the patient was bitten by her dog on the index finger but did not seek medical attendance because there were no signs of infection. Solely, a small sensory deficit of the affected finger was reported. The patient then presented with sudden abdominal cramps, bilious vomiting, epigastric pain, pyrexia up to 40 °C and shivering.

Upon admission to the regional hospital, the clinical examination revealed a tachycardic, hypotensive, tachypneic, and febrile patient with a tense abdomen and two small skin lesions without any signs of infection on her index finger. An abdominal ultrasound showed a dilated small-bowel with pendular peristalsis, ascites, and gallbladder wall thickening.

For suspected abdominal infection, an empiric antibiotic treatment with piperacillin/tazobactam was started after collecting blood cultures. Hence, the patient was immediately transferred to our tertiary teaching hospital.

**Fig. 1:**
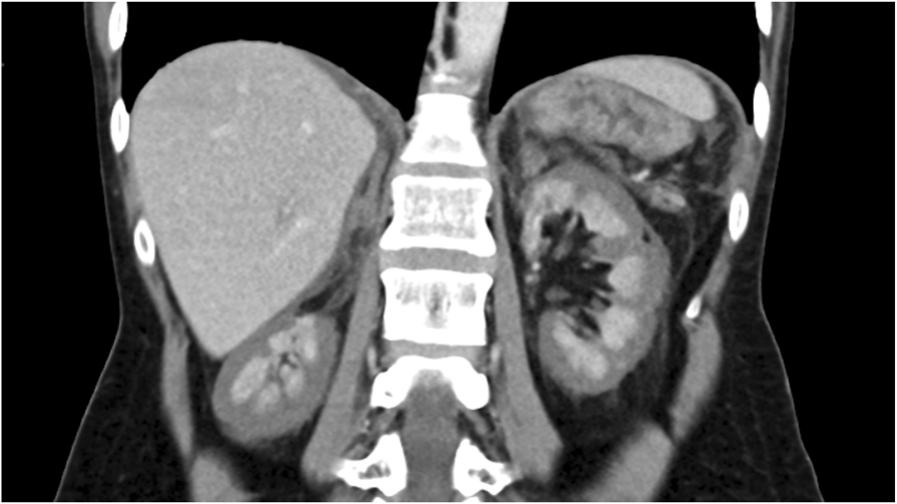
Contrast-enhanced abdominal CT scan, with a bilateral hypo-perfused renal cortex and an adequately perfused renal medulla = “reverse rim sign”. This finding is pathognomonic for acute renal cortical necrosis

Our hospital’s initial laboratory work-up revealed signs of infection, coagulopathy, acute kidney injury, and severe lactic acidosis with normal liver/pancreatic parameters (Table 2). The contrast-enhanced CT scan showed a reactive wall enhancement of the gallbladder, ascites, and bilateral ARCN by the “reverse rim sign” (Fig. 1). Due to a high suspicion of cholecystitis causing abdominal sepsis, the patient underwent open cholecystectomy. However, interoperative findings and histological examination did not support this diagnosis.

**Table 2:** Laboratory results on admission

Laboratory results on admission		
	Result	Reference
Leucocyte count (× 10^9^/l)	3.87	3.50–10.00
C-reactive protein (mg/l)	156	< 10
Hemoglobin (g/l)	111	120–160
Thrombocytes (×10^9^/l)	14	150–450
International Normalized Ratio	2.2	< 1.2
Creatinine (mmol/l)	133	42–80
Estimated glomerular filtration rate	42	> 90
CKD - EPI^a^ (ml/min/1.73/m^2^)		
pH^b^	7.23	7.38–7.42
Base excess^b^	−11.2	> − 5
Lactate^b^ (mmol/l)	8.7	< 1.8
Bicarbonate^b^ (mmol/l)	15.1	21–26

^a^ *CKD-EPI* Chronic Kidney Disease Epidemiology Collaboration [25]

^b^ arterial blood gas

**Fig. 2:**
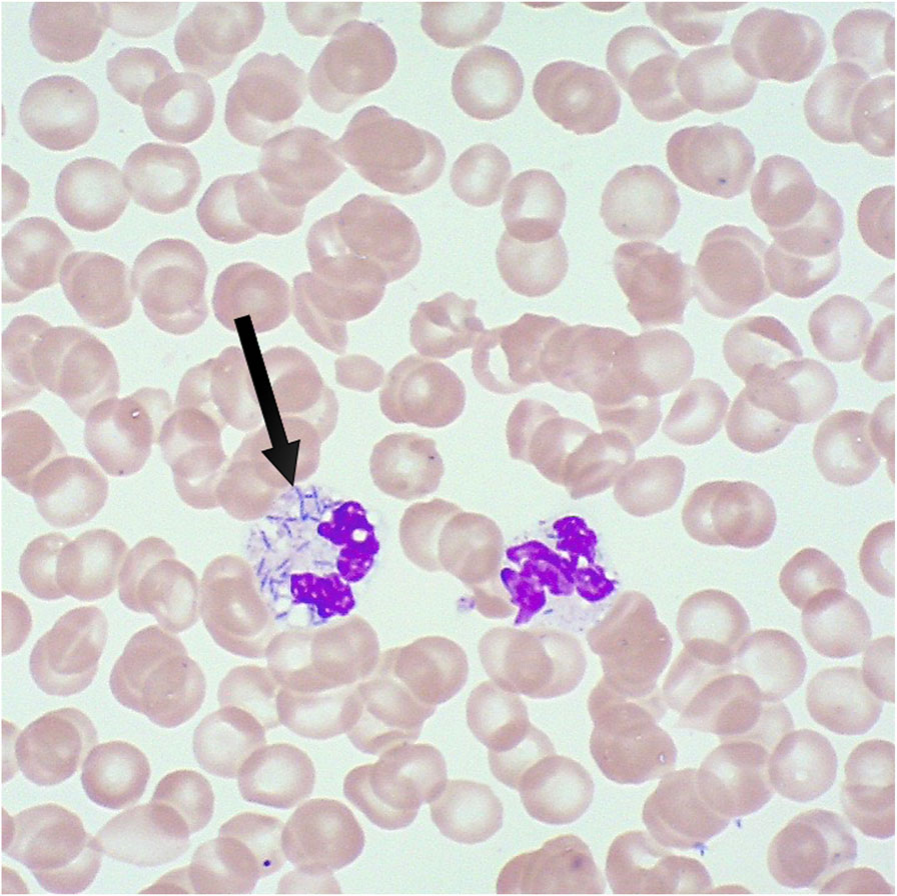
Peripheral blood smear with May-Grünwald-Giemsa stain. Polymorphonuclear leukocyte with intracellular rod-shaped bacteria (black arrow ); image kindly provided by *Health Center Fricktal*, Rheinfelden, Switzerland)

Postoperatively, the disseminated intravascular coagulation (DIC) worsened, and transfusion of coagulation factors was inevitable, calling for liver packing due to uncontrolled bleeding from the surgical site. Further clinical manifestations of the DIC were profuse bleeding from puncture sites and drainages as well as purpura fulminans. The clinical course was further complicated by a critical hemodynamic instability with profound hypotension and septic cardiomyopathy requiring inotropes. Shortly after admission to intensive care, the laboratory reported identification of rod-shaped bacteria directly from the first peripheral blood smear done in the regional hospital (Fig. 2), suggesting a high pathogen load. Later, blood cultures also revealed Gram-negative rods in Gram staining, which did not grow using standard culture conditions. Together with the clinical information about a dog bite, this raised suspicion for *C. canimorsus*. Bacterial growth was finally achieved on chocolate agar incubated under micro-aerophilic conditions and identified as *C. canimorsus* by MALDI-TOF mass spectrometry. *C. canimorus* was sensitive to penicillin (MIC = 0.004) and ceftriaxone (MIC = 0.023). The clinical course was further complicated by permanent hemodialysis and extensive acral necrosis, requiring amputation of several fingers and both thighs. After hemodynamic stabilization and prolonged weaning, the patient could be transferred to the ward after 6 weeks of intensive care and is currently making good progress in rehabilitation.

## Discussion and conclusions

To our knowledge, this is the first published case of a *C. canimorsus* infection complicated by bilateral ARCN with the rare CT-finding of a “reverse rim sign” (Fig. 1) [4–6] *C. canimorsus* associated disseminated intravascular coagulation may lead to microthrombi in renal glomeruli resulting in permanent kidney failure. *C. canimorsus* is usually susceptible to all beta-lactam antibiotics (including penicillin) and clindamycin. International guidelines suggest amoxicillin-clavulanate as prophylactic therapy after a dog bite [26]. The adjunction of beta-lactamase inhibitors seems reasonable, as beta-lactamases have been isolated in *Capnocytophaga spp* [27].

Concerning this rare pathogen, there is mainly anecdotal evidence in the form of case reports [7–11] and scoping reviews [12, 13] of published cases implicating a significant publication bias. Well-designed prospective research is warranted to gain further knowledge regarding this chameleon-like disease.

In conclusion, immunocompromised patients should be informed about the potential severe sequelae of animal bites and instructed to seek immediate medical advice, although bite wounds are minor or initial symptoms might be lacking. As proposed in the literature, physicians must initiate a pre-emptive antibiotic treatment in any wounds inflicted by cats and dogs in asplenic patients and should strongly consider pre-emptive treatment in other forms of immunosuppression (e.g., alcoholism) also [26, 28]. However, if ARCN is a new pathophysiologic entity associated with *C.canimorsus* infections or a result of the symptomatic DIC cannot be obtained from a single case.

## Data Availability

Not applicable.

## Abbreviations

*C. canimorsus*: *Capnocytophaga canimorsus*
CT: Computed tomography
ARCN: Acute renal cortical necrosis
DIC: Disseminated intravascular coagulation
MALDI-TOF: Matrix-assisted laser desorption/ionization time-of-flight mass spectrometry
MIC: Minimal inhibitory concentration
DF-2: Dysgonic fermenter-2
*Capnocytophaga spp*: *Capnocytophaga species*
